# 25-Hydroxyvitamin D status, vitamin D intake, and skin cancer risk: a systematic review and dose–response meta-analysis of prospective studies

**DOI:** 10.1038/s41598-020-70078-y

**Published:** 2020-08-04

**Authors:** Yahya Mahamat-Saleh, Dagfinn Aune, Sabrina Schlesinger

**Affiliations:** 1 CESP, Fac. de médecine - Univ. Paris-Sud, Fac. de médecine - UVSQ, INSERM, Université Paris Saclay, 94 805 Villejuif, France; 2 Inserm U1018, Gustave Roussy, 114 rue Edouard Vaillant, 94805 Villejuif, France; 3 Department of Epidemiology and Biostatistics, School of Public Health, Imperial College, London, UK; 4 Department of Nutrition, Bjørknes University College, Oslo, Norway; 5 Department of Endocrinology, Morbid Obesity and Preventive Medicine, Oslo University Hospital Ullevål, Oslo, Norway; 6 Institute for Biometrics and Epidemiology, German Diabetes Center, Leibniz Center for Diabetes Research At Heinrich Heine University, Düsseldorf, Germany

**Keywords:** Risk factors, Cancer prevention

## Abstract

Sun exposure is a major environmental risk factor for skin cancers and is also an important source of vitamin D. However, while experimental evidence suggests that vitamin D may have a protective effect on skin cancer risk, epidemiologic studies investigating the influence of 25-hydroxyvitamin D (25(OH)D) level and/or vitamin D intake on skin cancer risk are conflicting. A systematic review and dose–response meta-analyses of prospective studies was conducted to clarify these associations. Relevant studies were identified by searching the PubMed database up to 30th August 2019. Random effects dose–response meta-analyses were used to estimate summary relative risks (SRRs) and 95% confidence intervals (CIs). Overall, thirteen prospective studies were included. Circulating level of 25(OH)D was associated with higher risks of melanoma (SRR (95% CI) per 30 nmol = 1.42 (1.17–1.72)) and keratinocyte cancer (KC) (SRR (95% CI) per 30 nmol/L = 1.30 (1.13–1.49)). The SRR (95% CI) per 30 nmol/L increase in 25(OH) D level was 1.41 (1.19–1.67), and 1.57 (0.64–3.86), for basal cell carcinomas (BCCs) and squamous cell carcinomas (SCCs), respectively. However, while we found that vitamin D intake (from diet, supplemental and total) was not associated with risks of melanoma and SCC, vitamin D intake was associated with slightly increased BCC risk, albeit with no heterogeneity across skin cancer type. This meta-analysis suggests positive associations between circulating 25(OH)D level and risk of melanoma and KC, however, this finding is most likely confounded by sun exposure. We found no associations between vitamin D intake skin cancers, except positive associations with BCC risk.

## Introduction

Skin cancers are the most common type of malignancies in Caucasian populations^1^ and their incidence has risen worldwide over the past decades^2–4^. Melanoma is the most lethal form of skin cancer^5^, with highest incidence rates observed in Australia and New Zealand, followed by Northern America and Europe, and the lowest rates in Asian and African populations^1^. Although keratinocyte cancers (KCs), namely basal cell carcinoma (BCC) and squamous cell carcinoma (SCC), have low mortality rate, they have relevant impact on quality of life and healthcare costs^6,7^. The incidence of skin cancer has been expected to increase over the coming decades due to the increasing intensity of ultraviolet (UV) radiation received at the Earth's surface, which is caused by ozone depletion and global warming^8^. Established risk factors for skin cancer include sun exposure^9^^,^ which is currently the only factor on which prevention can be based, pigmentary traits^10^^,^ and family history of skin cancer^11^. However, some dietary factors, such as an antioxidant and anti-inflammatory-rich diet, have been suggested to prevent skin cancer^12,13^, whereas associations between vitamin D and skin cancer have produced a controversial debate in scientific and public communities.

Vitamin D is a fat-soluble vitamin that occurs in two natural forms, ergocalciferol (vitamin D2) and cholecalciferol (vitamin D3). These forms may be found in some food groups (e.g. fish, dairy products and cereal products), fortified foods (e.g. some dairy and cereal products), or dietary supplements. Most vitamin D is synthesized by exposure to ultraviolet radiation (UVR) in the skin, where 7-dehydrocholesterol is converted into vitamin D3^14^. Vitamin D2 and D3 are then metabolized in the liver into 25-hydroxyvitamin D (25(OH)D), which represents the major circulating form and reflects vitamin D status^15^. Besides its known role in maintaining bone health^16^^,^ vitamin D is involved in numerous biologic functions including anti-proliferative, anti-angiogenic and modulation of the immune system^17^. Emerging evidence suggests that vitamin D plays also a protective role against several types of cancer^18–21^. However, findings from epidemiologic studies investigating the association between vitamin D and risk of skin cancer remain inconsistent^22^. Several studies have indeed addressed the association between vitamin D and skin cancer risk^22,23^ which has been studied by exploring intakes of dietary vitamin D (from food or supplements), or circulating levels of 25(OH)D (a biomarker of vitamin D status reflecting both intake and synthesis related to sun exposure). Regarding 25(OH)D levels, most of the studies suggested an increased risk of melanoma and KC^24,25^ and some studies reported inverse^26,27^ or null associations^28,29^. While a recent meta-analysis based on four studies observed no association between high serum levels of 25(OH)D and melanoma risk, high 25(OH)D levels have been reported to be associated with increased risk of KC, particularly of BCC^30^. Nevertheless, this meta-analysis was limited by a small number of cases (392 melanoma cases and 768 KC cases) as well as by the lack of dose–response and subgroup analyses stratified by study characteristics, which were not possible due to small numbers of studies. Since the publication of this meta-analysis, several prospective studies have been published on vitamin D status and skin cancer^31–34^, and no previous meta-analysis has investigated the potential nonlinear dose–response relationship between 25(OH)D status and skin cancer risk. This could be useful for evaluating the balance between the potential benefits and risks for skin cancer prevention. In addition, previous epidemiologic studies have also yielded inconsistent results regarding the influence of dietary vitamin D on the risk of skin cancer^22,23^; some studies suggested that a high intake of dietary vitamin D may protect against skin cancer development^27,35^, while other studies found null results^35,36^. Caini and colleagues observed no statistically significant association for the highest versus lowest intake of vitamin D in relation to skin cancer^30^. In contrast, since then, new cohort studies on vitamin D intake and skin cancer have been published^32,34^ and no dose–response meta-analysis has been published to our knowledge.

Therefore, we conducted a systematic review and dose–response meta-analysis of prospective studies to investigate the association between vitamin D exposure (from diet, supplements and circulating level) and risk of skin cancer, including melanoma and KC (BCC and SCC).

## Methods

A systematic review following the guidelines for Meta-Analyses and Systematic reviews for Observational Studies^37^ and the PRISMA guidelines^38^ was conducted.

### Search strategy for study identification

A systematic search using several databases, such as PubMed, Embase, CAB Abstracts, ISI Web of Science, up to September 20th 2018, was performed by several reviews for eligible studies as part of the Continuous Update Project of the World Cancer Research Fund (WCRF-CUP) at Imperial College London. The protocol used for the search strategy can be accessed at https://www.wcrf.org/sites/default/files/skin-cancer-protocol.pdf. However, since all the relevant studies were initially identified by the PubMed search, we have changed the protocol and searches were updated to 30th August 2019 using the same search strategy only in the PubMed database. We additionally conducted the search in Google Scholar using the same specific key terms, and no new relevant articles were identified compared to PubMed search. In addition, we searched the reference lists of the relevant publications, reviews and meta-analysis for further studies.

### Selection criteria

We included in this meta-analysis prospective cohort or nested case–control studies investigating the association between either 25(OH)D level or vitamin D intake (dietary, supplemental, and total) and risk of melanoma or KC. Estimates of the relative risk (RR) (such as hazard ratios, risk ratios or odds ratios) with the 95% confidence intervals (CIs) had to be available in the publication, and for the dose–response analysis, a quantitative measure of intake or level of 25(OH)D, the total number of cases and person-years or non-cases had to be available in the publication. If several articles were published using the same study population on the topic, the one with the largest number of cases was selected^34,36,39^. Case–control studies, ecological studies, case reports, reviews and editorials were excluded from the meta-analysis. Studies which focused on the relationship between vitamin D exposure and survival from melanoma, recurrence or prognostic factor for melanoma (e.g. Tumor thickness, ulceration) were also excluded. The present study focused only on vitamin D exposure and risk of primary skin cancer.

### Data extraction

For each relevant study included in this meta-analysis, the following information was extracted by two reviewers: first author’s last name, publication year, country where the study was conducted, study name, study design, follow-up period, sex, age, number of cases, case ascertainment, exposure assessment, outcome, comparison, RRs and 95% CIs and adjustment factors (Table S1 and S2).

### Risk of bias

To assess the risk of bias and quality of the included studies, we used the Cochrane risk of bias tool ROBINS-I, which grades studies on a scale from critical risk of bias to low risk of bias due to confounding, selection, outcome and exposure assessment, classification as well as missing data^40^.

### Statistical methods

Dose–response and highest versus lowest meta-analyses were conducted to summarize the associations between vitamin D status or intake and risk of skin cancer by using random-effects models that consider both within study and between-study variation^41^. We used the method described by Greenland and Longnecker^42^ for the dose–response analysis to compute the trend from the correlated RRs and 95% CIs across categories of exposure when not provided in the publications. This method required the distribution of person-years, cases, median exposure, RRs, and 95% CIs for at least three categories. For studies that did not provide the distribution of person-years or the number of cases per categories, we estimated the distribution by dividing total person-years or case by the number of categories. The median of the exposure in each category was used if provided in the articles, and if not reported, the midpoint of the upper and lower boundaries was estimated as range in each category. When the highest and lowest categories were open-ended or had extreme upper or lower values, we used the width of the adjacent interval to estimate the upper and lower boundaries for the category. For studies that reported plasma or serum 25(OH)D level in ng/ml^24,26,32^, we converted the data to nmol/L by multiplying the concentration in ng/ml by 2.5, and for studies of vitamin D intake^32,43^, data in µg/day were divided by 0.025 to convert the data to IU/day.

For studies that reported results separately for BCC and SCC^24,25^, we combined the results by the Hamling procedure to obtain an overall estimate for KC^44^. The dose–response meta-analyses were conducted in increments of 30 nmol/L for 25(OH)D level^45^ and 100 IU/day for vitamin D intake^46^ based on the previous published studies^45,46^.

To explore the shape of the association between vitamin D levels and/or dietary vitamin D and incidence of skin cancer, we conducted non-linear dose–response meta-analysis using restricted cubic splines with 3 knots at 10th, 50th, and 90th percentiles of the distribution^47,48^. We also conducted subgroup and meta-regression analyses to investigate potential sources of heterogeneity by study characteristics such as sex, duration of follow-up, geographic location, risk of bias and adjustment for confounding factors. Statistical heterogeneity between studies was assessed by the Cochran Q test and the I^2^ statistic^49^. Small-study effects, such as publication bias, were assessed by visual inspection of funnel plot and with Egger’s test^50^^,^ and the results were considered to indicate potential small-study bias when *P* values were < 0.10. Sensitivity analyses excluding one study at a time were conducted to clarify whether the results were simply due to one large study or a study with an extreme result. Stata version 14 software (Stata Corp, College Station, TX) was used for the statistical analyses.

## Results

Overall, 284 of 27 546 identified articles were retrieved for full text review (Supplementary Fig. 1). Thirteen cohort studies^24–26,28,29,31–34,43,51–53^ were eligible for inclusion. Of these, ten studies were included in the meta-analysis of 25(OH)D level and skin cancer^24–26,28,29,31–33,51,53^ and four studies were included in the meta-analysis of vitamin D intakes from diet and supplement and skin cancer risk^32,34,43,52^ (Table S1 and S2). Seven studies were from North America^24,26,28,29,32,34,43^, five from Europe^31,33,51–53^, and one from Australia^25^. Eight studies were rated with moderate risk of bias^24,25,28,32,34,43,51,53^ and five with serious risk of bias^26,29,31,33,52^ (Table S3).

**Figure 1:**
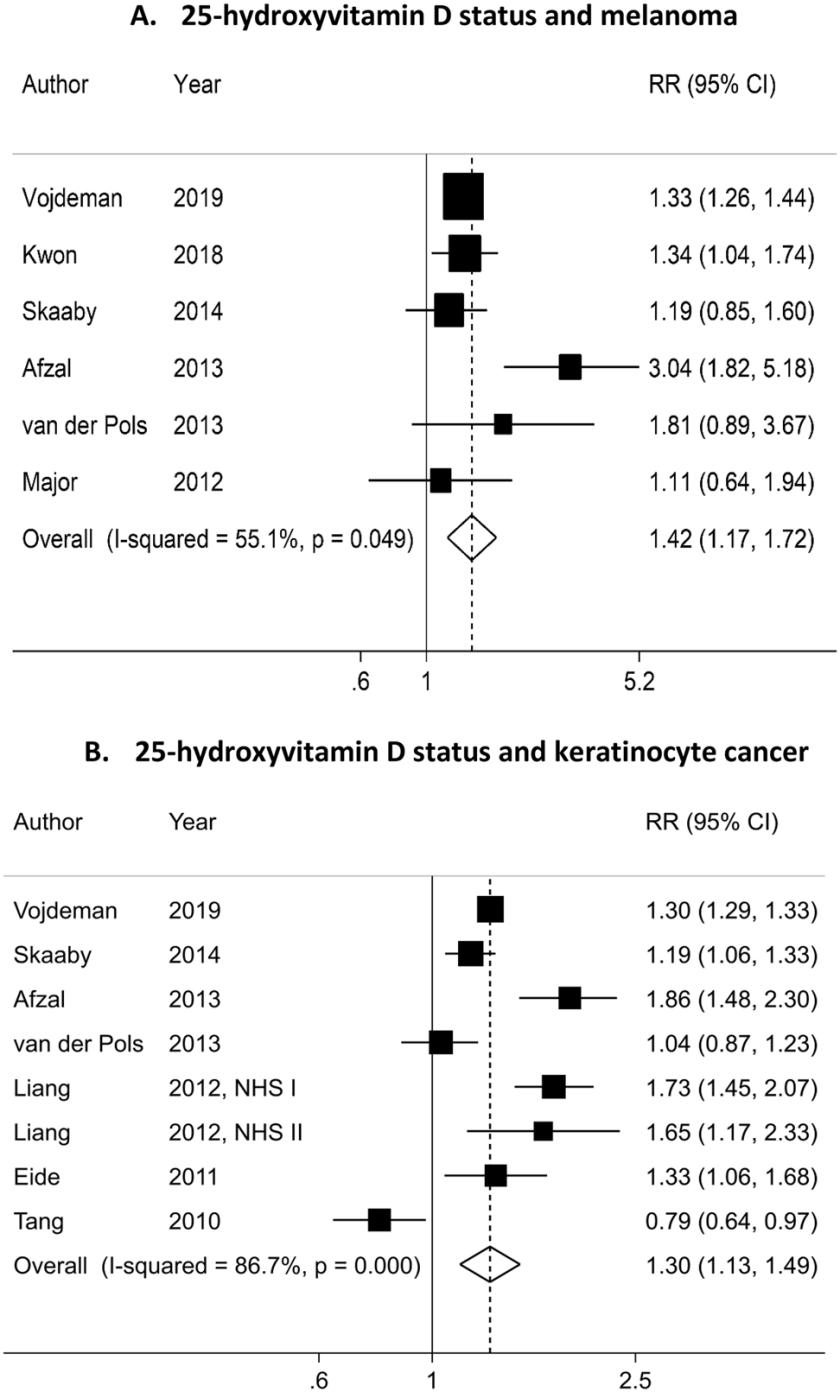
Dose–response meta-analysis of each 30 nmol/L increase in circulating 25-hydroxyvitamin D and the risk of skin cancer.

### Circulating level of 25(OH)D

**Figure 2:**
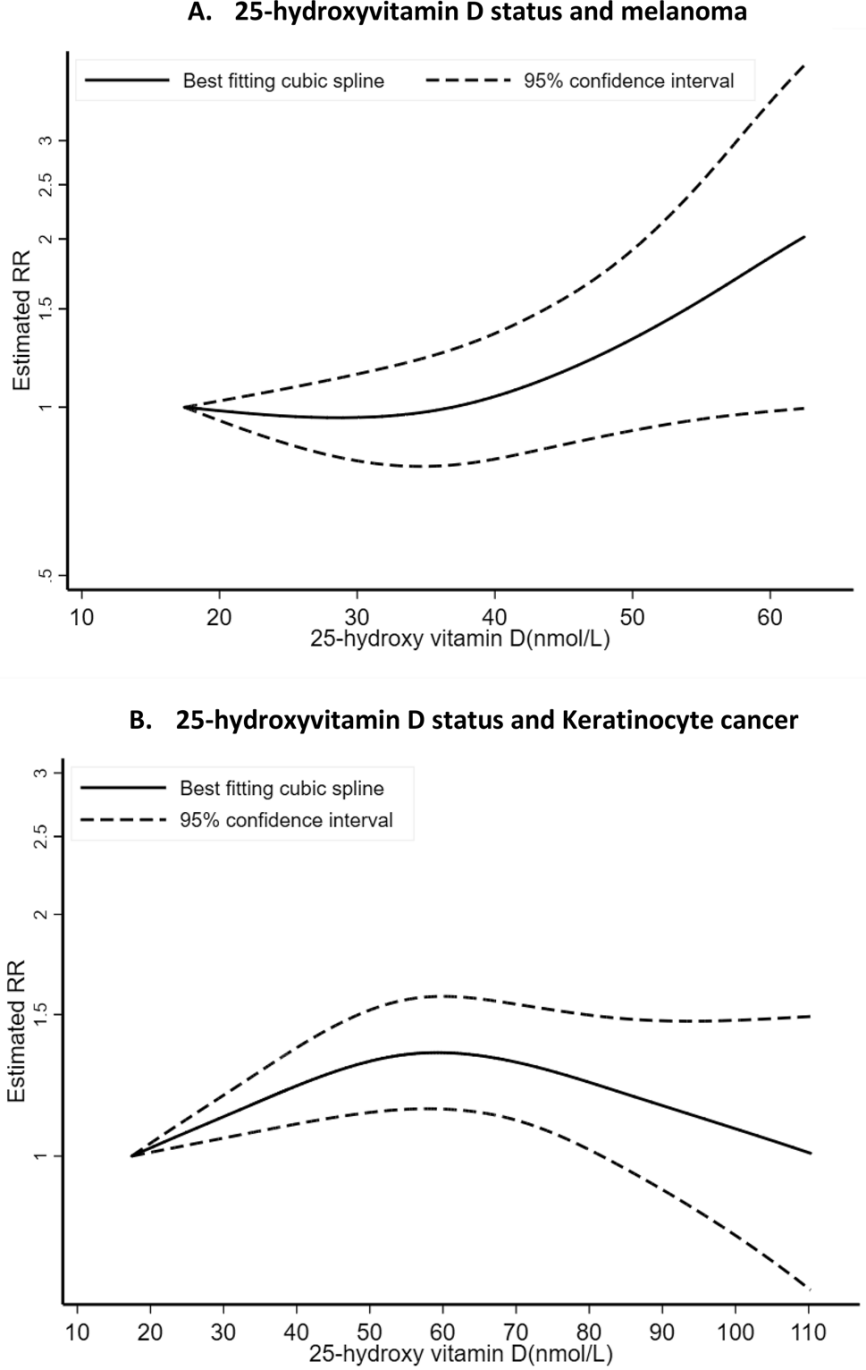
Non-linear dose–response relation between circulating 25-hydroxyvitamin D and skin cancer risk.

Six studies^25,31–33,51,53^ investigating the association between 25(OH)D level and risk of melanoma were included in the dose–response meta-analysis with a total of 1,644 cases among 241,893 participants. We observed a positive association between 25(OH) D level and melanoma risk (SRR per 30 nmol/L increment = 1.42; 95% CI = 1.17–1.72, I^2^ = 55%; P_heterogeneity_ = 0.05) (Table 1, Fig. 1A). There was no evidence of publication or small-study bias (*P* value Egger’s test = 0.45). Because of differences in the 25 (OH)D levels in the reference category among the studies, we could not fit an interpretable nonlinear model. However, when we excluded one study with the highest 25(OH)D level in the reference category^32^, we found no evidence of a nonlinear dose–response association (P_nonlinearity_ = 0.08) (n = 3 studies) (Fig. 2A). In the highest versus lowest meta-analyses (960 cases, five studies)^25,31,32,51,53^, a high 25(OH)D level was positively associated with melanoma risk (SRR = 1.60; 95% CI = 1.18–2.17, I^2^ = 0%, P_heterogeneity_ = 0.45) (Table 2, Figure S2A).

**Table 1:** Subgroup analyses of circulating 25-hydroxyvitamin D level and skin cancer risk.

	Vitamin D in blood, per 30 nmol/L									
	Melanoma					Keratinocyte cancer				
	n	RR (95% CI)	I^2^ (%)	P_within_^a^	P_between_^b^	n	RR (95% CI)	I^2^ (%)	P_within_^a^	P_between_^b^
All studies	6	1.42 (1.17–1.72)	55.1	0.05		8	1.30 (1.13–1.49)	86.7	< 0.0001	
Sex										
Men	1	1.11 (0.64–1.94)			0.52	1	0.79 (0.64–0.97)			0.01
Women	1	1.34 (1.04–1.74)				2	1.71 (1.46–2.01)	0.0	0.81	
Men and women	4	1.57 (1.14–2.17)	72.0	0.01		5	1.29 (1.15–1.46)	78.8	0.001	
Geographical location										
USA	1	1.34 (1.04–1.74)			0.42	4	1.31 (0.89–1.92)	91.1	< 0.0001	0.01
Europe	4	1.45 (1.08–1.96)	71.2	0.02		3	1.37 (1.17–1.59)	84.0	0.002	
Australia	1	1.81 (0.89–3.67)				1	1.04 (0.87–1.24)			
Duration of follow-up										
< 10 years	2	1.33 (1.25–1.42)	0.0	0.94	0.40	2	1.02 (0.63–1.67)	95.4	0.0001	0.48
≥ 10 years	4	1.61 (1.01–2.58)	71.2	0.02		6	1.42 (1.17–1.72)	83.5	0.0001	
Number of cases*										
Cases < 500	3	1.24 (0.96–1.60)	0.0	0.52	0.54	2	1.23 (0.99–1.52)	87.0	< 0.0001	0.04
Cases ≥ 500	3	1.57 (1.15–2.15)	78.8	0.01		6	1.53 (1.08–2.17)	90.1	0.0001	
Risk of bias										
Low	0				0.20					
Moderate	4	1.66 (1.09–2.52)	66.9	0.03		4	1.52 (1.13–2.06)	87.2	< 0.0001	0.02
Serious	2	1.32 (1.24–1.41)	0.0	0.50		4	1.15 (0.97–1.36)	87.5	< 0.0001	
Adjustment for confounders										
Age										
Yes	6	1.42 (1.17–1.72)	55.1	0.05	NA	8	1.30 (1.13–1.49)	86.7	< 0.0001	
No	0					0				
Sex										
Yes	4	1.57 (1.14–2.17)	72.0	0.01	0.79	5	1.29 (1.15–1.46)	78.8	0.001	0.94
No	2	1.30 (1.03–1.64)	0.0	0.55		3	1.30 (0.75–2.26)	94.1	< 0.0001	
Season										
Yes	3	1.60 (1.03–2.48)	79.0	0.01	0.48	5	1.37 (1.02–1.83)	91.3	< 0.0001	0.96
No	3	1.33 (1.25–1.42)	0.0	0.57		3	1.23 (1.06–1.42)	68.7	0.04	
Sun exposure										
Yes	3	1.34 (1.08–1.67)	0.0	0.57	0.99	3	1.42 (0.98–2.06)	88.5	< 0.0001	0.40
No	3	1.55 (1.08–2.25)	80.0	0.01		5	1.24 (1.05–1.47)	88.4	< 0.0001	
Hair color										
Yes	0				NA	2	1.71 (1.46–2.01)	0.0	0.81	0.01
No	6	1.42 (1.17–1.72)	55.1	0.05		6	1.21 (1.04–1.40)	87.7	< 0.0001	
Skin color										
Yes	1	1.81 (0.89–3.67)			0.40	1	1.04 (0.87–1.23)			0.02
No	5	1.40 (1.14–1.72)	61.7	0.03		7	1.34 (1.16–1.56)	87.0	< 0.0001	
Family history of skin cancer										
Yes	1	1.34 (1.04–1.74)			0.98	0				
No	5	1.48 (1.13–1.94)	64.1	0.03		8	1.30 (1.13–1.49)	86.7	< 0.0001	
Physical activity										
Yes	3	1.60 (1.03–2.48)	79.0	0.01	0.48	3	1.20 (0.80–1.80)	93.5	< 0.0001	0.05
No	3	1.33 (1.25–1.42)	0.0	0.57		5	1.36 (1.17–1.58)	78.0	0.001	
Smoking										
Yes	2	1.86 (0.74–4.65)	88.9	0.003	0.32	3	1.20 (0.80–1.80)	93.5	< 0.0001	0.05
No	3	1.33 (1.25–1.42)	0.0	0.77		5	1.36 (1.17–1.58)	78.0	0.001	
BMI										
Yes	4	1.48 (1.04–2.11)	70.6	0.02	0.65	3	1.20 (0.80–1.80)	93.5	< 0.0001	0.05
No	2	1.33 (1.25–1.43)	0.0	0.40		5	1.36 (1.17–1.58)	78.0	0.001	
Alcohol intake										
Yes	1	1.19 (0.87–1.63)			0.45	1	1.19 (1.06–1.33)	86.7	< 0.0001	0.13
No	5	1.50 (1.18–1.90)	0.62	0.32		7	1.32 (1.11–1.57)	88.1	< 0.0001	
Education										
Yes	2	1.28 (1.05–1.56)	0.0	0.56	0.63	1	1.19 (1.06–1.33)	86.7	< 0.0001	0.13
No	4	1.63 (1.09–2.45)	71.3	0.01		7	1.32 (1.11–1.57)	88.1	< 0.0001	

RR, summary relative risk; CI, confidence interval.

I^2^ (%) is a measure of the proportion of the heterogeneity attributed to between study variation rather than due to chance. I^2^-values of 25%, 50% and 75% indicates low, moderate and high between study heterogeneity, respectively.

^a^P-value for heterogeneity among the studies within each cancer type.

^b^P-value for between subgroup or category heterogeneity generated from meta-regression analysis.

**Table 2:** Summary results of vitamin D exposure and skin cancer risk, dose–response and high vs low and meta-analysis.

	Melanoma				Basal cell carcinoma				Squamous cell carcinoma			
	n	RR (95% CI)	I^2^ (%)	P_within_^a^	n	RR (95% CI)	I^2^ (%)	P_within_^a^	n	RR (95% CI)	I^2^ (%)	P_within_^a^
Circulating 25-hydroxyvitamin D levels (per an increase of 30 nmol/L)	6	1.42 (1.17–1.72)	55.1	0.05	4	1.41 (1.19–1.67)	44.4	0.15	3	1.57 (0.64–3.86)	88.4	0.0001
Highest vs. lowest level of vitamin D status	5	1.60 (1.18–2.17)	0.0	0.45	5	1.82 (1.49–2.21)	0.0	0.56	4	1.80 (0.64–5.04)	81.4	0.001
Vitamin D Intake (per an increase of 100 IU/day)												
Dietary vitamin D	3	1.01 (0.99–1.03)	0.0	0.71	3	1.04 (1.02–1.06)	9.8	0.33	2	1.02 (0.97–1.07)	0.0	0.69
Supplemental vitamin D	3	1.00 (0.96–1.03)	0.0	0.93	2	1.02 (1.00–1.03)	36.7	0.21	2	0.98 (0.95–1.01)	0.0	0.47
Total vitamin D	3	1.01 (0.99–1.02)	0.0	0.99	2	1.02 (1.00–1.03)	77.7	0.03	2	0.99 (0.97–1.01)	0.0	0.71
Highest vs. lowest vitamin D intake												
Dietary vitamin D	4	1.09 (0.93–1.27)	0.0	0.47	2	1.13 (1.08–1.18)	0.0	0.85	2	1.14 (0.95–1.36)	41.3	0.20
Supplemental vitamin D	3	1.03 (0.86–1.23)	0.0	0.73	2	1.07 (1.03–1.12)	0.0	0.41	2	0.95 (0.83–1.10)	0.0	0.57
Total vitamin D	3	1.08 (0.92–1.26)	0.0	0.82	2	1.10 (1.05–1.15)	0.0	0.34	2	1.02 (0.89–1.17)	0.0	0.78

RR summary relative risk; CI confidence interval.

I^2^ (%) is a measure of the proportion of the heterogeneity attributed to between study variation rather than due to chance. I^2^-values of 25%, 50% and 75% indicates low, moderate and high between study heterogeneity, respectively.

^a^P-value for heterogeneity among the studies within each cancer type.

A total of eight studies^24–26,29,31,33,53^ were included in the dose–response meta-analysis of 25(OH) D level and risk of KC, including 7,485 cases and 249,108 participants. We found that 25(OH) D levels were associated with higher risk of KC (SRR per 30 nmol/L increment = 1.30; 95% CI = 1.13–1.49, I^2^ = 86%, P_heterogeneity_ = 0.001) (Table 1, Fig. 1B). There was no evidence of publication bias with Egger’s test (*P* = 0.94). There was indication for a nonlinear dose–response association between 25 (OH) D level and KC (P_nonlinearity_ = 0.01) (n = 3 studies) (Fig. 2B). The strongest relative risk for KC was observed at a level of approximately 60 nmol/L of 25(OH) D with a weaker association beyond this level. In addition, a positive association was observed in the highest versus lowest analysis (SRR = 1.64; 95% CI = 1.11–2.43, I^2^ = 86.0%, P_heterogeneity_ = 0.0001)^24–26,29,31,53^ (2,440 cases, seven studies) (Figure S2B).

**Figure 3:**
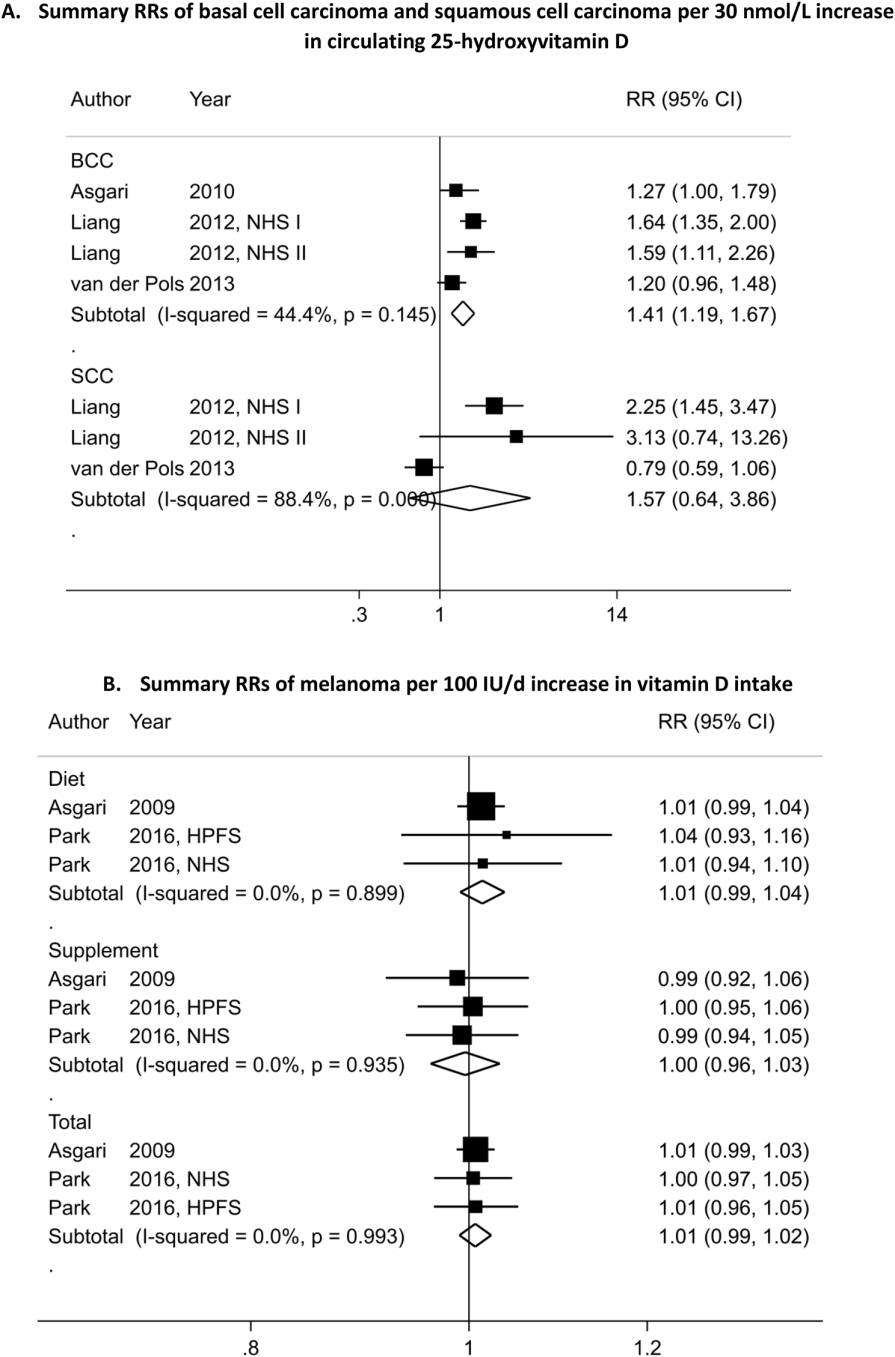
Dose–response meta-analyses on circulating 25-hydroxyvitamin D, dietary, supplemental and total vitamin D intake, and skin cancer risk.

**Figure 4:**
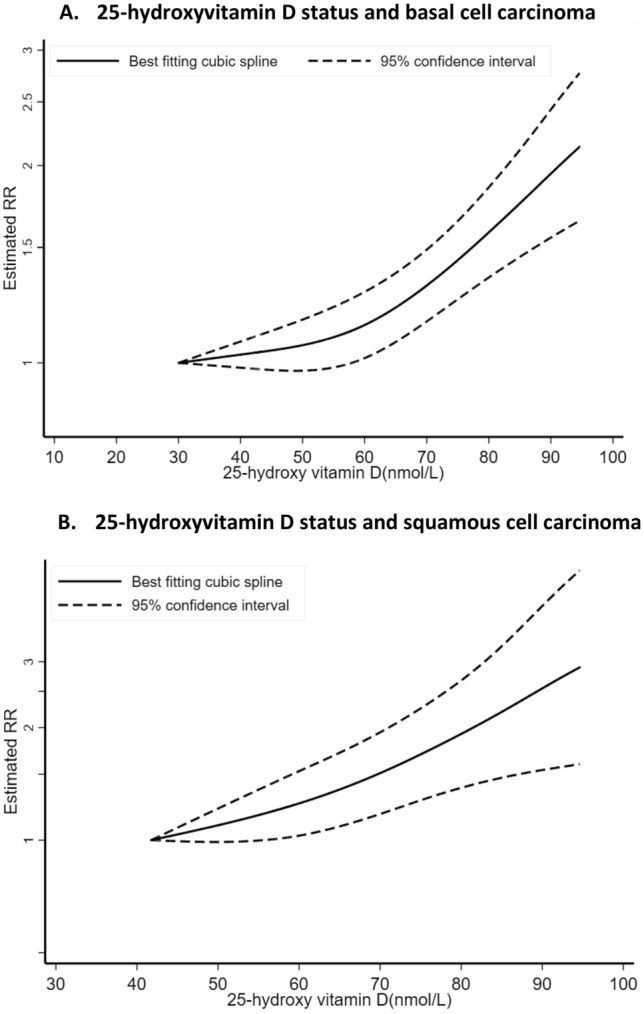
Non-linear dose–response relation between circulating 25-hydroxyvitamin D and basal cell and squamous cell carcinoma risks.

In meta-analyses stratified by KC type, we observed a statistically significant positive association between 25(OH)D level and BCC risk (SRR = 1.41; 95% CI = 1.19–1.67, I^2^ = 44%, P_heterogeneity_ = 0.15) for an increase of 30 nmol/L (1,030 cases and four studies) (Table 2, Fig. 3A)^24,25,28^. The SRR per 30 nmol/L increase in 25(OH)D level was 1.57 (95% CI = 0.64–3.86, I^2^ = 88.4%, P_heterogeneity_ = 0.001) for SCC, suggesting a positive but not precisely estimated, expressed by the wide 95% CIs (251 cases and three studies)^24,25^, and no heterogeneity across skin cancer types was detected (P_heterogeneity_ = 0.53) (Table 2, Fig. 3A). While, there was indication of nonlinear dose–response of BCC and 25(OH)D (P_nonlinearity_ = 0.004) (n = 3 studies) (Fig. 4A) with a stronger increase in risk at the higher level of 25(OH)D (around the value of 60 nmol/L), there was no evidence of nonlinearity dose–response for SCC (P_nonlinearity_ = 0.28) (n = 2 studies) (Fig. 4B). The SRR for the highest versus lowest meta-analysis was 1.82 (95% CI = 1.49–2.21) and 1.80 (95% CI = 0.64–5.04) for BCC and SCC, respectively (Table 2, Figure S2C).

### Vitamin D intake

**Figure 5:**
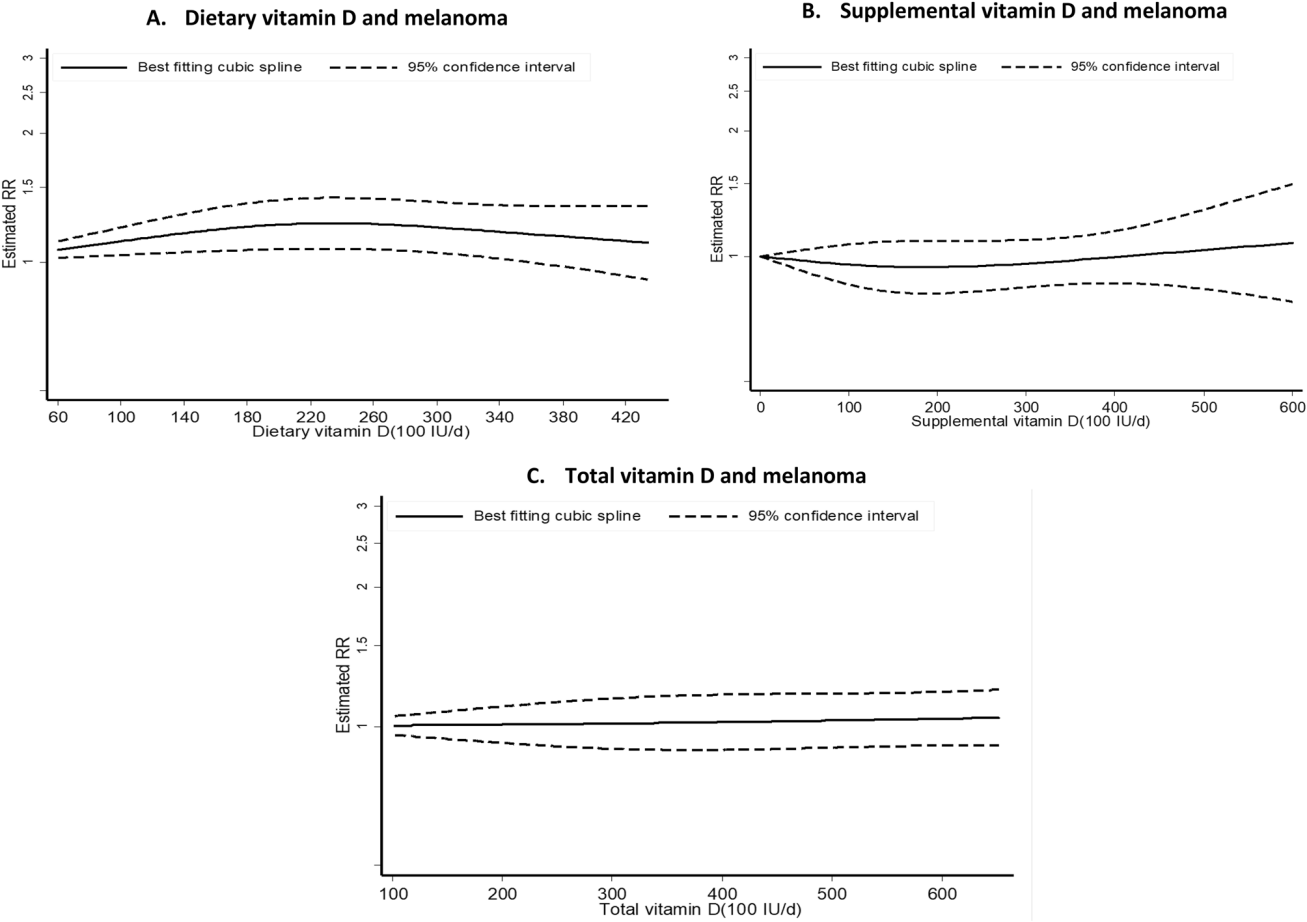
Non-linear dose–response relation between vitamin D intake (from diet, supplement and total) and melanoma risk.

Three studies^34,43^ investigated the association between vitamin D intake and melanoma risk with a total of 2,493 cases among 183,445 participants. No significant associations were observed. The summary RR per 100 IU/day increment was 1.01 (95% CI = 0.99–1.03, I^2^ = 0%, P_heterogeneity_ = 0.71) for dietary vitamin D, 1.00 (95% CI = 0.96–1.03, I^2^ = 0%, P_heterogeneity_ = 0.93) for supplemental vitamin D and 1.01 (95% CI = 0.99–1.02, I^2^ = 0%, P_heterogeneity_ = 0.99) for total vitamin D (Table 2, Fig. 3B). There was no significant evidence for small study effects for all exposure. While there was indication of a non-linear dose–response association between dietary vitamin D intake and melanoma risk (P_nonlinearity_ = 0.03) (Fig. 5A), no evidence of non-linear dose–response was found for supplemental (P_nonlinearity_ = 0.44) (Fig. 5B) and total vitamin D intake (P_nonlinearity_ = 0.91) (n = 3 studies) (Fig. 5C). Similar results were found when the highest intake was compared to the lowest intake (Table 2).

**Figure 6:**
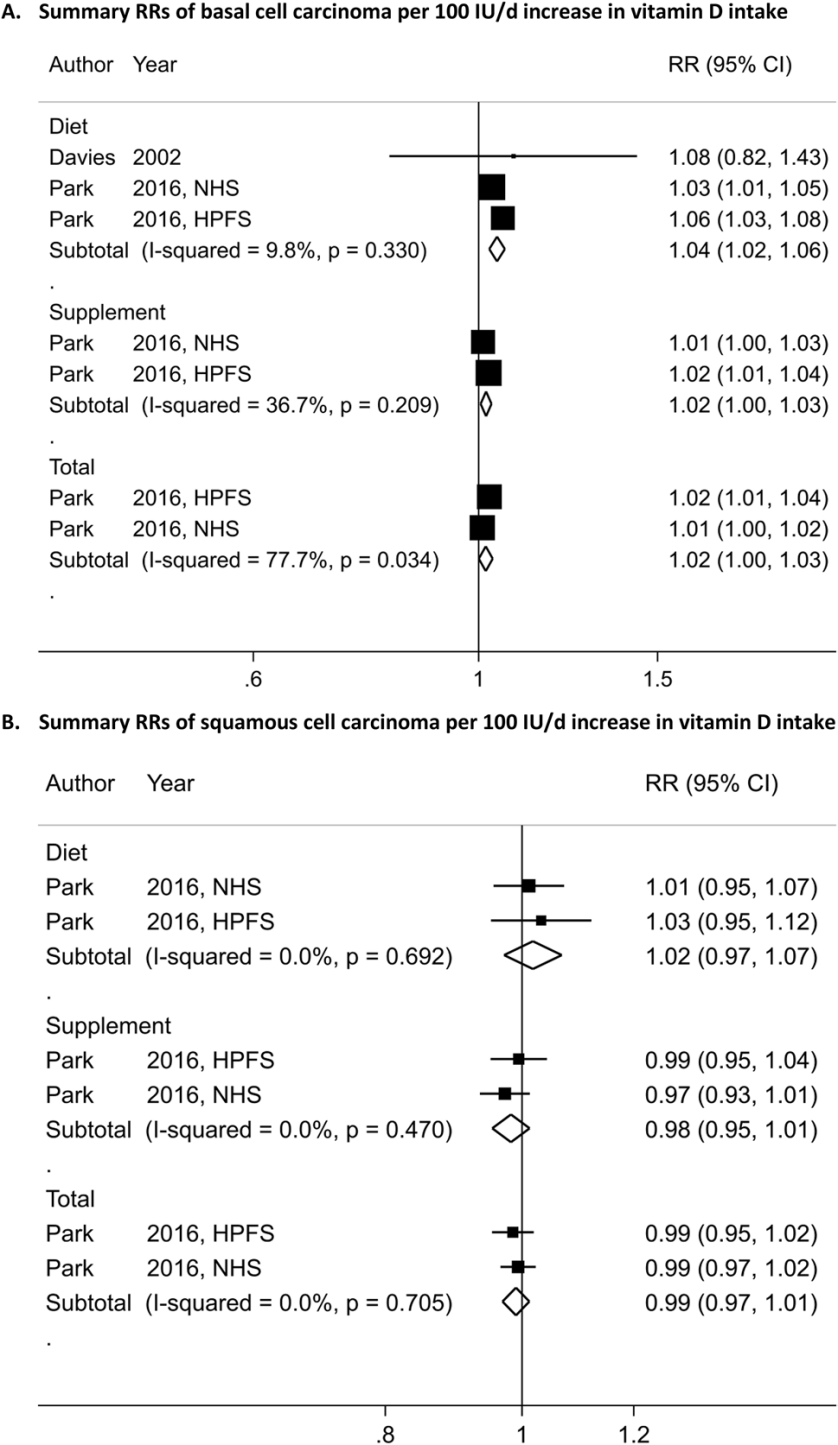
Dose–response meta-analysis of each 100 IU/day increase in vitamin D intake (from diet, supplement and total) and the risk of skin cancer.

Three^34,52^ and two prospective studies^34^ reported on the relationship between vitamin D intake and risks of BCC (20,949 cases, 114,363 participants) and SCC, respectively (2,329 cases, 114,116 participants). While we observed positive associations between vitamin D intake (SRR per 100 IU/day increment = 1.04 (95% CI = 1.02–1.06) for dietary, 1.02 (95% CI = 1.00–1.03) for supplemental and 1.02 (95% CI = 1.00–1.03) for total vitamin D) and risk of BCC (Fig. 6A), we found no significant associations between vitamin D intake and risk of SCC (SRR per 100 IU/day increment was 1.02 (95% CI = 0.97–1.07) for dietary, 0.98 (95% CI = 0.95–1.01) for supplemental and 0.99 (95% CI = 0.97–1.01) for total vitamin D) (Fig. 6B). Here again, there was no heterogeneity detected across skin cancer type (all P_heterogeneity_ was > 0.30). Non-linear dose–response analysis could not be done for vitamin D intake and BCC and SCC due to insufficient studies.

### Subgroup analyses

Associations between vitamin D status and melanoma risk persisted in most subgroup analyses including analyses by sex, duration of follow-up, number of cases, geographical location, risk of bias and adjustment for confounding factors, including sun exposure. While there was no evidence of between-subgroup heterogeneity, heterogeneity within subgroup analyses was still present (Table 1). In sensitivity analyses excluding the most influential studies, the summary RR for 30 nmol /L increment of 25(OH)D and melanoma risk was ranged from 1.33 (95% CI = 1.24–1.41) when excluding the Copenhagen City Heart Study^53^ to 1.51 (95% CI = 1.11–2.07) when excluding the Copenhagen database (CopD) study^33^ (Figure S3A).

In stratified analyses according to sex, the association between 25(OH)D level and KC risk was positive among women, whereas an inverse association was observed among men with heterogeneity detected across sex (P_heterogeneity_ = 0.01); however only one study was available for men. There was heterogeneity between subgroup analyses of KC including those by geographical location with stronger associations among the European studies (P_heterogeneity_ = 0.01), by number of cases with stronger associations for studies with more than 500 cases (P_heterogeneity_ = 0.04), and by risk of bias with higher association for studies with moderate compared to those with serious risk of bias (P_heterogeneity_ = 0.02). In addition, there was heterogeneity by adjustment for hair color (P_heterogeneity_ = 0.02), skin color (P_heterogeneity_ = 0.01), adjustment for physical activity (P_heterogeneity_ = 0.05), smoking status (P_heterogeneity_ = 0.05) and BMI (P_heterogeneity_ = 0.05). However, in the remaining subgroup analyses, between-subgroup heterogeneity was not significant, and the positive associations persisted in most of them, although the results were more imprecise. In sensitivity analyses excluding the most influential studies for KC, the summary RR per 30 nmol /L increment ranged from 1.24 (95% CI = 1.07–1.42) when excluding the Copenhagen City Heart Study^53^ to 1.38 (95% CI = 1.22–1.55) when excluding the Osteoporotic Fractures Study^32^ (Figure S3B). Subgroup analyses of vitamin D intakes and risks of melanoma and KC could not be done because of the limited number of studies.

## Discussion

This meta-analysis of thirteen prospective studies suggests that vitamin D status was associated with greater risks of melanoma and KC. Linear dose–response meta-analysis revealed that each 30 nmol /L increment in 25(OH)D was respectively associated with 42%, 30% and 41% increase risks of developing melanoma, KC and BCC. In non-linear dose–response meta-analysis, the association was significant for KC, with higher relative risk observed at a level of approximately 60 nmol/L of 25(OH) D, and a weaker association beyond this level. However, while dietary, supplemental and total intakes of vitamin D were not associated with melanoma and SCC risks, these latter were slightly associated with higher risk of BCC, although with no heterogeneity across skin cancer type. Similar findings were observed in high versus low meta-analysis.

Previous research has suggested that high plasma/serum 25(OH)D concentration could have a protective effect against several chronic diseases^54,55^, including cancer risk^46,56,57^. Animal and human studies have indeed demonstrated that vitamin D status may influence some cancers, such as colon, stomach, kidney as well as skin through down regulation of cell growth^58,59^ and modulation of the immune system^60^. Vitamin D is mainly synthesized in the skin after exposure to UV radiation, and it has been estimated 80–90% of vitamin D are from sun exposure and the remainder amount is from the diet and supplements intake^61^. However, although UV radiation is recognized as a major skin carcinogen, the same spectrum of UV exposure which can lead to DNA damage in skin cells also induce vitamin D synthesis^62,63^. This latter has been suggested to have anticancer properties in normal and skin cancer cells^64–66^. Consequently, this has led to a strong debate among scientific and public communities about the balance between the potential benefits and risks of UV-induced vitamin D production and skin cancer prevention. A meta-analysis, based on 4 case–control or cohort studies published between 2009 and 2013, suggested no statistically significant association between serum 25-(OH)D levels and the risk of melanoma, while a positive association was found with KC risk^30^. Unfortunately, this previous meta-analysis did not conduct dose–response meta-analyses or subgroup analyses by confounding factors that are critical to consider due to the conflicting results among published studies to date. The dose–response curve for vitamin D status and skin cancer provides insights regarding an optimal value for vitamin D status, which can be identified for skin cancer prevention, and for proposing an optimal level.

To our knowledge, this is the first meta-analysis to explore the linear and non-linear dose–response association between vitamin D status and the risk skin cancer, including only prospective studies, and the first to investigate subgroup analyses of the associations. Additionally, this is an updated meta-analysis to investigate the highest versus lowest serum or intake of vitamin D in relation to skin cancer^30^. In the present meta-analysis, we found that high vitamin D status was associated with greater risks of melanoma and KC. We found some evidence of a non-linear dose–response association between circulating 25(OH)D levels and KC risk, with the strongest summary relative risk observed at a level of approximately 60 nmol/L of 25(OH) D, which may be considered as insufficient vitamin D status. While levels of 25(OH)D below 50 nmol/L have suggested to be associated with severe vitamin D deficiency, levels between 50 and 74 nmol/L have been described as moderate vitamin D deficiency or insufficiency, and levels between 75–99 are considered as sufficient. Serum 25(OH)D levels between 100 and 150 nmol/L are indicated as adequate and healthy^67,68^. However, consistent with our findings, a recent prospective study suggested that higher levels of 25(OH)D was significantly associated with a higher incidence of skin cancer, with the hazard ratio seemed to peak around a vitamin D status of 80 nmol/L in the non-linear trend analysis^33^. Because of the limited number of studies included in our non-linear dose–response meta-analyses, mainly due to the missing information on the range of vitamin D levels, further studies should aim to clarify the shape of the dose–response relationship.

The positive association between 25(OH)D levels and skin cancer risk is likely due to increased UV exposure causing both higher vitamin D levels and skin cancer risk. The current findings lend to support previous meta-analysis, published in 2014, that estimated a SRR of 1.64 (95% CI: 1.02–2.65) for the relationship between high serum 25(OH) D levels and KC risk, and of 1.46 (95% CI: 0.60–3.53) for the association with melanoma risk^30^. UV exposure induces both DNA lesion and immune suppression through production of reactive oxygen species which can alter the cellular redox equilibrium leading to premature skin aging and lipid peroxidation^69,70^. DNA damage caused by both UVB and UVA, together with UV-induced immune suppression contributes to the development of skin cancer^71^. Although, UVA and UVB are both implicated in skin cancer development^72^, studies reported that UVA plays a larger role in melanoma, whereas keratinocyte cancers are mainly related to UVB exposure^73^, which known to induce vitamin D production. Several studies suggested that UVB exposure induces genetic alterations, which in turn promote skin carcinogenesis^74–76^. UVB is mainly absorbed within the epidermis and upper dermis and is associated with skin burning and cause damage to keratinocytes in vitro^77^. However, while UVB radiation contributes to increase skin cancer risk, ecological studies reported an inverse correlation between solar UVB doses and other cancer risk^78–81^. Another hypothesis to explain the positive association between vitamin D status and skin cancer risk may be attributed to genetic factors. Several studies have addressed the issue of whether single nucleotide polymorphisms (SNPs) of the vitamin D receptor (VDR) gene are associated with the risk of developing different types of cancer^82,83^, and several VDR variants have been investigated in relation to skin cancer risk^84,85^, with three meta-analyses supporting a positive association, particularly with FokI and BsmI^86–88^. Since vitamin D exerts a function of engagement of its receptor VDR, it is likely that some SNPs of the VDR gene affect the ability of interacting with its ligand, which ultimately would lead to different levels of the biologic activity of vitamin D and increase skin cancer risk. Additional research is required to confirm the potential role of VDR gene in skin cancer incidence and to explore their interaction with sun exposure. A recent study based on the UK Biobank data, using a methodically robust Mendelian randomization, found no evidence of a causal association between genetic determinants of vitamin D concentrations and risk of melanoma^89^. However, while a Mendelian randomization study found no evidence for a causal association between genetically predicted vitamin D concentration and overall cancer risk^90^^,^ another one reported a weak evidence for linear causal associations between genetic determinants of circulating vitamin D levels and risk of several cancers cancers^91^.

Given the contrasting and confounding effects of sun exposure and others factors such as pigmentary trait and dietary vitamin D on vitamin D status, it is, to date, difficult to examine an independent influence of vitamin D status on skin cancer risk. In our subgroup analysis, the positive association between circulating 25(OH)D levels and risk of melanoma was observed across all subgroup analyses. For KC, the positive association was stronger among studies conducted in Europe, in those with more than 500 cases and with moderate risk bias and in those adjusted for hair color, whereas association were not significant among studies adjusted for skin color, physical activity and BMI. In addition, our findings suggested a moderate and a substantial heterogeneity among studies for melanoma and KC, respectively. Part of heterogeneity for melanoma appeared to be attributed to study duration of follow-up; number of cases; risk of bias and some adjustment for confounders. This heterogeneity could also be related to melanoma localization or histologic type. Previous studies suggested the model of heterogeneity for melanoma by showing distinct etiologies for different body sites and tumor types^92,93^. However, this aspect has been largely overlooked in previous studies. Although there was a high level of heterogeneity across studies investigating vitamin D status in relation to SCC, no heterogeneity was detected for BCC, suggesting that difference in KC type may lead to important study heterogeneity.

Nevertheless, anti-proliferative and pro-apoptotic properties of vitamin D have indeed been demonstrated in animal and in vitro studies^94,95^, and 1,25(OH)D has been shown to regulate multiple signaling pathways involved in differentiation, inflammation, invasion, angiogenesis and metastasis^17^. Recently, a pooled analysis of 25 studies showed that lower vitamin D levels were associated with higher Breslow thickness and mortality rates in patients with melanoma^96^. Several studies suggested that higher vitamin D levels may confer better prognosis from melanoma including Breslow thickness^30,97^. UV exposure has also been reported to be associated with a better prognosis and survival rate in several cancers sites^98^^,^ and some authors proposed that these may include melanoma^99,100^, which could be explained by UV exposure–induced high serum levels of vitamin D and lead to a better prognosis. Holiday sun exposure before melanoma diagnosis has been reported to be associated with lower thickness and the exposure after melanoma diagnosis was also associated with reduce melanoma recurrence^101^. Furthermore, increasing evidence has been suggested that circulating levels of vitamin D may play a protective role against several types of cancer such a bladder^102^ and colorectal^103^. Also, a recent pooled analysis of two randomized controlled trials (RCTs) and a prospective cohort found that higher 25(OH)D levels were inversely associated with breast cancer risk with levels ≥ 60 ng/ml ((≥ 150 nmol/L) being most protective ^104^.

Regarding intake of vitamin D, our finding suggested that intake of vitamin D from diet or supplement was not associated with melanoma and SCC risk, whereas there was a weak positive association with BCC risk, although with no heterogeneity across skin cancer type. Two studies from the NHS and HPFS were indeed included in these analyses. As mentioned by the authors, the positive association was mainly attributed to the vitamin D-rich foods such as fish and cereal which were associated with BCC after adjustment for several known risk factor of skin cancer. It could be argue that arsenic present in fish and breakfast cereals, including rice, may be responsible for the positive association. These findings were based on few studies, and thus, the results need to be interpreted with caution. Despite this research, mounting evidence reported a beneficial effect of vitamin D supplementation in reducing cancer incidence and mortality^105,106^. A meta-analysis of RCT suggested that evidence is stronger for cancer mortality rather than cancer incidence^107^.

Our meta-analysis has several strengths including the prospective studies, the relatively large sample size, and the linear and nonlinear dose–response analyses. As many previous published meta-analysis, the current analysis has several limitations. First, it is important to notice that all included studies have considered only a single baseline measure of 25 (OH)D, which may not necessarily represent long-term status. Also, most of studies lacked sensitivity analyses excluding skin cancer cases diagnosed within 2, 4, or 6 years of blood draw in order to assess whether reverse causation could have influenced the findings. Thus, we could not investigate a subgroup analysis by time of vitamin D measurement. However, one measurement of plasma 25(OH)D has been suggested to reflect long-term sun exposure and seemed to predict skin cancer risk^24^. In support of this supposition, previous study have reported a correlation of 0.70 for repeated measures of plasma 25(OH)D within individuals over time, suggesting that a single measurement is a reasonable proxy for long-term levels of 25(OH)D^108^. Further limitations of this meta-analysis include risk of bias of the primary studies, including potential measurement error in the assessment of exposure, residual confounding, especially regarding sun exposure. In addition, some of our meta-analyses were based on a low number of primary studies, and thus, subgroup and analyses by e.g. sun exposure level, season and pigmentary traits were not possible or relied on small numbers of studies.

In conclusion, our finding suggests that high vitamin D status was associated with increased risks of melanoma and KC. Given that 25 (OH)D level is mainly from sun exposure, higher risk of skin cancer may be confounded by sun exposure, data for which is lacking in most studies. However, while we found that intakes of dietary or supplemental vitamin D were not associated with risk of melanoma and SCC, high intakes of vitamin D from diet and supplements were slightly associated with BCC risk, albeit with no heterogeneity across skin cancer. Overall, the current evidence suggests that unprotected sun exposure should be avoided in order to achieve high vitamin D status, and that an adequate amount of vitamin D should be obtained from a healthy diet.

## Supplementary information

Supplementary Information.
