# Supplementary material for: 25-Hydroxyvitamin D status, vitamin D intake, and skin cancer risk: a systematic review and dose–response meta-analysis of prospective studies
Source: Sci Rep. 2020 Aug 4;10:13151. doi: 10.1038/s41598-020-70078-y (PMC7403339; doi:10.1038/s41598-020-70078-y)
Supplement: Supplementary file 1 — Supplementary Information. [file 41598_2020_70078_MOESM1_ESM.docx]

**25-hydroxyvitamin D status, vitamin D intake, and skin cancer risk: A systematic review and dose-response meta-analysis of prospective studies**

**Authors:**

Yahya Mahamat-Saleh^1,2^, Dagfinn Aune^3,4,5^, Sabrina Schlesinger^6^

**Affiliations:**

^1^ CESP, Fac. de médecine - Univ. Paris-Sud, Fac. de médecine - UVSQ, INSERM, Université Paris Saclay, 94 805, Villejuif, France
^2^ Gustave Roussy, F-94805, Villejuif, France
^3^ Department of Epidemiology and Biostatistics, School of Public Health, Imperial College,
London, United Kingdom
^4^ Department of Nutrition, Bjørknes University College, Oslo, Norway
^5^ Department of Endocrinology, Morbid Obesity and Preventive Medicine, Oslo University Hospital Ullevål, Oslo, Norway
^6^ Institute for Biometrics and Epidemiology, German Diabetes Center, Leibniz Center for Diabetes Research at Heinrich Heine University, Düsseldorf, Germany.

***Corresponding author**: Yahya Mahamat-Saleh, Inserm U1018, Health across Generations Team, Gustave Roussy, Espace Maurice Tubiana, 114 rue Edouard Vaillant, F-94805 Villejuif Cedex, France; Tel: +33 1-42-11-56-45; Fax: +33 1 4211 4000; Email:[Yahya.MAHAMAT-SALEH@gustaveroussy.fr](mailto:Yahya.MAHAMAT-SALEH@gustaveroussy.fr)

| **Supplementary Table 1.** Prospective cohort studies of circulating 25-hydroxyvitamin D and skin cancer | | | | | | | | |
| --- | --- | --- | --- | --- | --- | --- | --- | --- |
| **First author, Publication year, study location** | **Study name, study design, characteristics** | **Cases/ Study size Follow-up (years)** | **Case ascertainment** | **Exposure assessment** | **Outcome** | **Comparison** | **Relative risk (95% confidence interval)** | **Adjustment factors/statistical methods used** |
| Skaaby T et al, 2014, Denmark | Pooled study: Monica10, Inter99, Health2006, Age: 18-71 years, M/W | 369 KCs; 55 melanoma / 12 204,  11.3 years | Cancer registry | IDS-SYS 25- Hydroxy Vitamin D method (Monica10); HPLC (Inter99); Cobas e411(Health 2006) | KC, all  KC, M  KC, W    Melanoma, all  Melanoma, M Melanoma, W | per 10 nmol/l  Q 1  Q 2  Q 3  Q 4  P_trend_  per 10 nmol/l  per 10 nmol/l  per 10 nmol/l  Q 1  Q 2  Q 3  Q 4  P_trend_  per 10 nmol/l  per 10 nmol/l | 1.06 (1.02-1.10)  1  1.18 (0.86–1.61)  1.35 (0.99–1.83)  1.43 (1.05-1.93)  0.015  1.06 (1.00-1.12)  1.06 (1.00-1.12)  1.06 (0.95-1.17)  1  0.52 (0.21–1.33)  1.24 (0.60–2.60)  1.18 (0.56-2.48)  0.275  1.04 (0.90-1.21)  1.07 (0.93-1.23) | study, sex, education, season during which blood was drawn, physical activity, smoking habits, alcohol intake, intake of fish, and BMI  Risk estimates by Cox proportional-hazards regression model |
| Afzal S et al, 2013, Denmark | The Copenhagen City Heart Study (CCHS), Prospective Cohort, Age: 20-100 years, M/W | 590 KCs;  78 melanoma / 10 060;  20.5 years | Danish cancer registry | DiaSorin LIAISON 25 (OH) vitamin D TOTAL assay | KC  Melanoma | per 10 nmol/l  Clinical categories (nmol/l)  ≤25  25–49.9  ≥50  P_trend_  Seasonally adjusted tertiles  T1  T2  T3  ≥100 vs. ≤25 nmol/l  per 10 nmol/l  Clinical categories (nmol/l)  ≤25  25–49.9  ≥50  P_trend_  Seasonally adjusted tertiles  T1  T2  T3  ≥100 vs. ≤25 nmol/l  Sun-exposed  sites (head and extremities)  per 10 nmol/l  unexposed  sites (trunk and other sites)  per 10 nmol/l | 1.23 (1.14-1.32)  1  2.60 (1.5-4.2)  5.04 (2.78-9.16)  3.10^–8^  1  2.10 (1.2-3.80)  4.02 (2.45–6.60)  2.10^–8^  5.28 (1.66-16.8)  1.45 (1.22-1.73)  1  1.60(0.96-7.10)  4.72 (0.96- 23.30)  0.02  1  6.30 (1.38–28.8)  6.30 (1.38–28.8)  0.03  9.58 (2.37-38.70)  1.58 (1.25–2.00)  1.24 (0.93–1.66) | Age, sex, BMI, income, occupational physical activity, calendar month of blood draw, cumulative tobacco consumption, physical intensity of leisure-time activities, running and cycling habits  Risk estimates by Cox proportional-hazards regression model |
| van der Pols JC et al,  2013, Australia | Nambour skin cancer study (NSCS),  Prospective analysis in adults who had participated in a skin cancer prevention trial (1992–1996) of daily sunscreen use and beta-carotene supplementation Age: 54 years, M/W | 300 BCC;  176 SCC;  17 melanoma / 1 191;  11 years | Questionnaires and skin examination with histological confirmation (100%) | LIAISON  25(OH)D assay | BCC  SCC  Melanoma | per 50 nmol/l  <75 nmol/l  ≥75 nmol/l  <50 nmol/l  ≥50 nmol/l  <50 nmol/l  ≥75 nmol/l  per 50 nmol/l  <75 nmol/l  ≥75 nmol/l  <50 nmol/l  ≥50 nmol/l  <50 nmol/l  ≥75 nmol/l  per 50 nmol/l  <75 nmol/l  ≥75 nmol/l  <50 nmol/l  ≥50 nmol/l  <50 nmol/l  ≥75 nmol/l | 1.35 (0.94-1.93)  1  1.51 (1.10-2.07)  1  1.38 (0.95-2.00)  1  1.74 (1.13-2.67)  0.68 (0.42-1.11)  1  0.67 (0.44-1.03)  1  0.78 (0.50-1.23)  1  0.61 (0.35-1.06)  2.70 (0.83-8.77)  1  2.71 (0.98-7.48)  1  1.53 (0.42-5.56)  1  2.75 (0.68- 11.17) | Age, sex, propensity to sunburn, skin colour, treatment allocation, elastosis neck, family history of skin cancer, freckling back, personal history of skin cancer before 1996, usual time spent outdoors  Risk estimates by logistic regression model |
| Liang G et al, 2012, USA | Nurses’ Health Study (NHS) and II, Nested Case Control,  Age: 25-55 years, W | **BCC**  510/ 4056 controls NHS and NHS II  387/ 1641 controls, NHS  123/ 2415 controls, NHS II  **SCC**  75/ 4056 controls, NHS and NHS II  67/ 1641 controls, NHS  8/ 2415 controls, NHS II | Self-reported medical history annually verified by medical records | Radioimmunoassay or chemiluminescence immunoassay | **BCC**  NHS I and NHS II  NHS I  NHS II  NHS I and NHS II, summer  spring and fall  winter  UVB flux  ≤113  >113  Light pigmentation  Dark pigmentation  **SCC**  NHS I and NHS II  NHS I  NHS II | Q1  Q2  Q3  Q4  P_trend_  ≤20.4 ng/ml  20.4-27.0 ng/ml  27.0-34.2 ng/ml  >34.2 ng/ml  P_trend_  ≤19.6 ng/ml  19.6-25.5 ng/ml  25.5-31.4 ng/ml  >31.5 ng/ml  P_trend_  Q1  Q2  Q3  Q4  P_trend_  Q1  Q2  Q3  Q4  P_trend_  Q1  Q2  Q3  Q4  P_trend_  Q1  Q2  Q3  Q4  P_trend_  Q1  Q2  Q3  Q4  P_trend_  Q1  Q2  Q3  Q4  P_trend_  Q1  Q2  Q3  Q4  P_trend_  Q1  Q2  Q3  Q4  P_trend_  ≤20.4 ng/ml  20.4-27.0 ng/ml  27.0-34.2 ng/ml  >34.2 ng/ml  P_trend_  ≤19.6 ng/ml  19.6-25.5 ng/ml  25.5-31.4 ng/ml  >31.5 ng/ml  P_trend_ | 1  1.17(0.86–1.58)  1.54(1.15–2.07)  2.07 (1.52-2.80)  <0.0001  1  1.18(0.83–1.68)  1.57(1.11–2.23)  2.28 (1.58-3.29)  <0.0001  1  1.20(0.67-2.13)  1.63(0.93-2.85)  1.93 (1.10-3.37)  0.01  1  0.68(0.37-1.27)  0.88(0.48-1.62)  0.93(0.51-1.71)  0.81  1  1.45(0.92-2.29)  2.10(1.35-3.28)  2.97 (1.90-4.63)  <0.0001  1  1.33(0.75-2.37)  1.44(0.81-2.57)  2.53 (1.36-4.72)  0.006  1  1.49(1.02–2.18)  1.81(1.23-2.67)  2.66(1.78-3.97)  <0.0001  1  0.80(0.47-1.34)  1.25(0.78-1.98)  1.52(0.94-2.46)  0.03  1  1.08(0.69-1.68)  1.81(1.18-2.78)  2.31(1.50-3.56)  <0.0001  1  1.26(0.83–1.91)  1.38(0.91–2.08)  1.85(1.21–2.85)  0.005  1  1.40(0.60-3.28)  2.81(1.29-6.12)  3.77 (1.70-8.36)  0.0002  1  1.49(0.61-3.66)  3.04(1.33-6.95)  3.96 (1.68-9.34)  0.0004  1  0.48(0.08-27.86)  2.62 (0.19-36.30)  4.95 (0.41-59.28)  0.15 | Age at blood collection, cohort, hair colour,  laboratory batch, number of sunburns, propensity to sunburn, season of blood draw, UVB flux, NHS and NHS II combined adjusted for cohort  Risk estimates by logistic regression model |
| Major JM et al, 2012,  Finland | Alpha-Tocopherol Beta-Carotene Cancer Prevention (ATBC), Nested Case Control,  Age: 50-69 years, Men Smokers | 92 melanoma/ 276 controls 18.2 years | Finnish cancer registry | LIAISON 25-OH Vitamin D Total Assay | Melanoma | Clinically-defined categories (nmol/l)  ≤24.9  25.00-37.49  37.50-49.99  ≥50  P_trend_  Season-adjusted residuals quartiles (nmol/l)  <3.12  3.12–3.49  3.50–3.89  ≥3.90  P_trend_ | 1  1.04 (0.52-2.12)  0.60 (0.25-1.44)  1.32 (0.64-2.72)  0.51  1  1.20 (0.55-2.64)  1.56 (0.74-3.28)  0.96 (0.43-2.17)  0.49 | Age at randomization, cholesterol, date of blood draw, height, propensity to sunburn, weight  Risk estimates by logistic regression model |
| Tang JY et al, 2010, USA | Osteoporotic Fractures in Men  (MrOS) Study, Nested Case Control, Age: 65- years, M, Elderly | 178 KC/ 930 controls | Self-reported | LC-mass spectroscopy | KC | ≤15.9 ng/ml  16-20.8 ng/ml  20.9-25.1 ng/ml  25.2-29.8 ng/ml  29.9-58.3 ng/ml  P_trend_  ≥32 vs. ≤31.9 ng/ml  ≥29.9 vs. ≤15.9 ng/ml (Q5 vs. Q1) | 1  0.94 (0.56–1.55) 0.93 (0.56–1.54)  0.86 (0.51–1.45)  0.54 (0.31-0.96  0.044  0.59 (0.34-1.01)  0.60 (0.37-0.98) | Age, BMI, cigarette smoking, clinic site, season of blood draw, outdoor walking activity  Risk estimates by logistic regression model |
| Asgari M et al, 2010, USA | Kaiser Permanente Northern California (KPNC), Nested Case Control, Age: 54.9 years, M/W | 220 BCC/ 220 controls;  8.74 years | Pathology reports | DiaSorin LIAISON 25(OH) Vitamin D Total Assay | BCC | clinical tertiles (ng/ml)  ≤9.9  10-<30  ≥30  P_trend_  Per 1 ng/ml  ≤14.69 ng/ml  14.70-20.06 ng/ml  20.07-24.67 ng/ml  24.68-29.78 ng/ml  >29.78 ng/ml  P_trend_ | 1  2.30 (0.70, 7.60)  3.61 (1.00- 13.10)  0.03  1.02 (1.00-1.05)  1  1.67 (0.84-3.34)  1.11 (0.51-2.43)  1.54 (0.70-3.37)  2.09 (0.95-4.58)  0.11 | BMI, educational level, history of cancer, smoking status, x-ray, sun exposure surrogates (hours of exercise and leisure activities)  Risk estimates by logistic regression model |
| Eide M et al, 2011, USA | Henry Ford Health System (HFHS), Prospective Cohort, Age: 65.9 years, M/W | 240 KCs;  191 BCC;  77 SCC / 3 223,  9.8 years | Pathology reports | Radioimmunoassay | KC  BCC  SCC  Less UV-Exposed,  KC  BCC  SCC | <19 ng/ml  19-24 ng/ml  25-30 ng/ml  ≥31 ng/ml  P_trend_  ≥15 vs. <15 ng/ml  ≥15 vs. <15 ng/ml  ≥15 vs. <15 ng/ml  ≥15 vs. <15 ng/ml  ≥15 vs. <15 ng/ml  ≥15 vs. <15 ng/ml | 1  1.3 (0.9-1.9)  1.4 (0.96-2.1)  1.60 (1.10-2.30)  0.02  1.80 (1.10-2.90)  1.70 (1.00-2.90)  1.70 (0.70-4.00)  2.2 (0.7-7.0)  3.2 (0.4-24.0)  1.7 (0.5-5.8) | Age, sex  Risk estimates by logistic regression model |
| Vojdeman et FJ al, 2019,  Denmark | The Copenhagen database (CopD),  Prospective cohort study,  Age: median 48.8 years, M/W | 5,045 KC;  684 melanoma/217,244 | Danish Cancer Registry | LIAISON 25(OH)D assay | KC  Melanoma | per 10 nmol/L  Men and women  Men  Women  per 10 nmol/L,  Men and women  Men  Women | 1.09 (1.09–1.10)  1.11 (1.09–1.12)  1.09 (1.08–1.10)  1.10 (1.08–1.13)  1.12 (1.08–1.17)  1.10 (1.07–1.13) | Age in 1-year intervals, sex, month of sampling, and comorbidity  Risk estimates by Cox proportional-hazards regression model |
| Kwon G et al,  2018,  USA | Women’s Health Initiative (WHI),  Nested case-control study, postmenopausal  women,  Age: 50-79 | 718 melanoma /718 controls,  9 years | Self-reported medical history annually verified by medical records | Liquid chromatographye mass  spectrometry (LC-MS) | Melanoma | ≤20.0 ng/mL  20.1-29.9 ng/mL  ≥30.0 ng/mL  P_trend_ | 1  1.79 (1.22-2.62)  1.59 (1.04-2.42)  0.01 | Age, BMI, education, multivitamin and calcium intake,  sun exposure history, history of skin cancer, physical activity, season  of blood draw, smoking, time spent outdoors in the summer both  in childhood and in adulthood, use of sunscreen, regional solar  irradiance (in langleys), and having a medical visit in the last year  Risk estimates by Cox proportional-hazards regression model |

Note: BCC, Basal-cell carcinoma; BMI, Body mass index, KC, Keratinocyte cancer; M, Men; SCC, Squamous-cell carcinoma; UV: Ultraviolet; W, women

| **Supplementary Table 2.** Prospective cohort studies of vitamin D intake (from diet and supplement) and skin cancer | | | | | | | | |
| --- | --- | --- | --- | --- | --- | --- | --- | --- |
| **First author, Publication year, study location** | **Study name, characteristics** | **Cases/ Study size Follow-up (years)** | **Case ascertainment** | **Exposure assessment** | **Outcome** | **Comparison** | **Relative risk (95% confidence interval)** | **Adjustment factors** |
| Asgari M et al, 2009, USA | The Vitamins and Lifestyle cohort study (VITAL), Prospective Cohort, Age: 50-76 years, M/W | 455 melanoma / 68 611, 6 years  420/  450/ | Cancer registry | Total FFQ | Melanoma | *Dietary*  *Supplement use, 10-year use of individual supplements*  none  Former/current vs.  None  10 μg/day  ≥15 μg/day  P_trend_  *Dietary (*μg/d)  0-3  3.0-4.7  4.7-7.1  >7.1-53  P_trend_  *Diet plus supplement (μg/d)*  0-5.10  >5.1–9.5  >9.5–14  >14-58  P_trend_ | 1  1.08 (0.82-1.43)  0  1.00 (0.71-1.40)  0.77 (0.34-1.72)  0.67  1  1.09 (0.80–1.48)  1.41 (1.04–1.90)  1.31 (0.94–1.82)  0.05  1  0.91 (0.68-1.22)  0.97 (0.73-1.30)  1.05 (0.79-1.40)  0.56 | Age, gender, education, 1-degree family history melanoma, personal history of KC, ever had moles removed, freckles between ages 10 and 20 years, had ≥3 severe sunburns between ages 10 and 20 years, natural red/blond hair between ages 10 and 20 years, and reaction to 1-h in strong sunlight; dietary and total intakes additionally adjusted for total energy intake  Risk estimates by Cox proportional-hazards regression model |
| Davies TW et al, 2002,  UK | European Prospective Investigation into Cancer and Nutrition–Norfolk (EPIC-Norfolk), Nested Case Control, Age: 65 (W), 67.8 (M), M/W | 109 BCC /247 controls | East Anglian Cancer Registry | Dietary Validated self-reported 7-day food diary | BCC | *Dietary*  Per 2.08 μg/day | 1.07 (0.85-1.35) | BMI, red hair colour, dietary component  Risk estimates by logistic regression model |
| Park SM et al,  2016,  USA | Nurses’ Health Study (NHS), and the Health Professionals Follow-up Study (HPFS), Prospective Cohort, Age:  NHS: 30-55, W  HPFS: 40–75, M | 20,840 BCC; 2,329 SCC; 1,320  Melanoma/114116  24-26 years | Self-report verified by medical records | Dietary Semi-quantitative FFQ | **BCC,**  NHS + HPFS  NHS  HPFS  **SCC,**  NHS + HPFS  NHS  HPFS  **Melanoma,**  NHS + HPFS  NHS  HPFS | *Total*  Q1  Q2  Q3  Q4  Q5  P_trend_  *Dietary (U/d)*  Q1  Q2  Q3  Q4  Q5  P_trend_  *Supplement (*IU/d)  None  1-99  100–199  200-399  ≥400  P_trend_  *Total*  124.8  210.9  304.5  437.4  638.2  P_trend_  *Dietary (U/d)*  97.6  145.0  185.2  233.6  314.4  P_trend_  *Supplement (IU/d)*  0  57.1  133.3  266.2  400.0  P_trend_  *Total (IU/d)*  156.0  253.5  363.2  519.9  775.3  P_trend_  *Dietary (IU/d)*  126.3  192.3  245.8  311.0  433.8  P_trend_  *Supplement (IU/d)*  0  56.0  133.3  251.2  400.0  P_trend_  *Total*  Q1  Q2  Q3  Q4  Q5  P_trend_  *Dietary*  Q1  Q2  Q3  Q4  Q5  P_trend_  *Supplement (IU/d)*  None  1-99  100–199  200-399  ≥400  P_trend_  *Total (IU/d)*  124.8  210.9  304.5  437.4  638.2  P_trend_  *Dietary (U/d)*  97.6  145.0  185.2  233.6  314.4  P_trend_  *Supplement (IU/d)*  0  57.1  133.3  266.2  400.0  P_trend_  *Total (IU/d)*  156.0  253.5  363.2  519.9  775.3  P_trend_  *Dietary (IU/d)*  126.3  192.3  245.8  311.0  433.8  P_trend_  *Supplement (IU/d)*  0  56.0  133.3  251.2  400.0  P_trend_  *Total*  Q1  Q2  Q3  Q4  Q5  P_trend_  *Dietary*  Q1  Q2  Q3  Q4  Q5  P_trend_  *Supplement (*IU/d)  None  1-99  100–199  200-399  ≥400  P_trend_  *Total (IU/d)*  124.8  210.9  304.5  437.4  638.2  P_trend_  *Dietary (U/d)*  97.6  145.0  185.2  233.6  314.4  P_trend_  *Supplement (IU/d)*  0  57.1  133.3  266.2  400.0  P_trend_  *Total (IU/d)*  156.0  253.5  363.2  519.9  775.3  P_trend_  *Dietary (IU/d)*  126.3  192.3  245.8  311.0  433.8  P_trend_  *Supplement (IU/d)*  0  56.0  133.3  251.2  400.0  P_trend_ | 1  1.03 (0.99-1.08)  1.08 (1.04-1.13)  1.10 (1.04-1.16)  1.10 (1.05, 1.15)  0.05  1  1.06 (1.01-1.11)  1.10 (1.05-1.16)  1.10 (1.06-1.15)  1.13 (1.08-1.18)  <0.001  1  1.03 (0.99-1.08)  1.06 (0.99-1.13)  1.05 (0.99-1.10)  1.07 (1.03-1.12)  0.03  1  1.02 (0.95-1.08)  1.08 (1.02-1.15)  1.13 (1.06-1.20)  1.12 (1.05-1.19)  <0.0001  1  1.03 (0.97-1.10) 1.13 (1.06-1.20) 1.11 (1.04-1.18) 1.13 (1.07-1.20) <0.001  1  1.02 (0.96-1.08) 1.03 (0.97-1.09) 1.07 (1.02-1.13) 1.09 (1.03-1.16) <0.001  1  1.05 (0.98-1.13)  1.08 (1.01-1.16)  1.06 (0.99-1.14)  1.07 (1.00-1.15)  0.14  1  1.09 (1.02-1.16) 1.07 (1.00-1.15) 1.10 (1.03-1.18) 1.12 (1.04-1.20) <0.01  1  1.05 (0.99-1.13) 1.10 (1.02-1.17) 1.02 (0.96-1.08) 1.05 (0.98-1.12)  0.27  1  1.04 (0.91-1.19)  1.07 (0.94-1.23)  0.84 (0.73-0.96)  1.02 (0.89-1.17)  0.30  1  1.21 (0.84-1.73) 1.04 (0.76-1.43) 1.09 (0.86-1.38) 1.14 (0.95-1.36) 0.41  1  0.98 (0.85-1.11) 0.94 (0.82-1.07) 0.92 (0.82-1.04) 0.95 (0.82-1.09)  0.26  1  1.04 (0.86-1.25)  1.04 (0.87-1.26)  0.83 (0.68-1.01)  1.00 (0.82-1.21)  0.33  1  1.45 (1.20-1.76) 1.23 (1.01-1.49) 1.23 (1.01-1.50) 1.25 (1.02-1.52) 0.43  1  1.04 (0.86-1.26) 0.96 (0.79-1.16) 0.98 (0.82-1.17) 1.00 (0.80-1.23) 0.70  1  1.04 (0.86-1.25)  1.11 (0.92-1.34)  0.85 (0.70-1.04)  1.04 (0.85-1.26)  0.61  1  1.00 (0.83-1.21) 0.89 (0.73-1.07)  0.97 (0.80-1.17)  1.04 (0.86-1.26)  0.66  1  0.91 (0.76-1.10) 0.92 (0.76-1.11) 0.88 (0.75-1.04) 0.92 (0.76-1.11) 0.24  1  1.12 (0.90-1.39)  1.05 (0.88-1.26)  1.04 (0.87-1.24)  1.09 (0.91-1.31)  0.71  1  1.16 (0.97-1.39)  1.17 (0.95-1.43)  1.26 (0.98-1.62)  1.06 (0.88-1.28)  0.55  1  1.05 (0.88-1.24) 0.93 (0.78-1.11) 0.95 (0.81-1.12) 1.04 (0.87-1.26) 0.97  1  1.24 (0.98-1.58)  1.10 (0.86-1.40)  1.09 (0.85-1.39)  1.15 (0.90-1.49)  0.74  1  1.18 (0.93-1.51)  1.29 (1.01-1.64)  1.43 (1.12-1.81)  1.05 (0.81-1.36)  0.56  1  0.97 (0.77-1.22) 0.89 (0.70-1.12) 0.93 (0.74-1.15) 1.08 (0.83-1.40) 0.83  1  0.99 (0.76-1.30)  1.01 (0.77-1.31)  0.99 (0.76-1.29)  1.03 (0.78-1.35)  0.85  1  1.14 (0.87-1.47) 1.04 (0.80-1.36) 1.10 (0.84-1.43) 1.08 (0.82-1.41) 0.76  1  1.15 (0.89-1.48) 0.97 (0.74-1.28) 0.98 (0.77-1.24) 1.01 (0.78-1.32) 0.78 | family history of melanoma , natural hair color, number of arm moles, skin reaction to sun exposure as a child/adolescent, number of lifetime blistering sunburns, average time spent in direct sunlight since high school, cumulative UV flux since baseline, body mass index, physical activity, smoking status, intakes of total energy, alcohol, and citrus intake. Analyze for women were also adjusted for menopausal status and postmenopausal hormone use  Risk estimates by Cox proportional-hazards regression model |
| Kwon G et al,  2018,  USA | Women’s Health Initiative (WHI),  Nested case-control study, postmenopausal  women,  Age: 50-79 | 718 melanoma /718 controls | Self-reported medical history annually verified by medical records | Questionnaire | Melanoma | *Dietary (IU/d)*  ≤400 IU  <400 IU | 1  0.80 (0.47-1.38) | Age, BMI, education, multivitamin and calcium intake,  sun exposure history, history of skin cancer, physical activity, season  of blood draw, smoking, time spent outdoors in the summer both in childhood and in adulthood, use of sunscreen, regional solar irradiance (in langleys), and having a medical visit in the last year  Risk estimates by Cox proportional-hazards regression model |

Note: BCC, Basal-cell carcinoma; BMI, Body mass index; FFQ, Food frequency questionnaire; KC, Keratinocyte cancer; M, Men; SCC, Squamous-cell carcinoma; UV: Ultraviolet; W, women

| **Supplementary Table 3.** Risk of bias judgements for each studies from the ROBINS-I | | | | | | | | |
| --- | --- | --- | --- | --- | --- | --- | --- | --- |
| **Author, Year** |  | | | | | |  | Overall judgement |
|  | **Bias due to confounding** | **Bias due to selection of participants** | **Bias due to exposure assessment** | **Bias due to misclassification during follow-up** | **Bias due to missing data** | **Bias due to measurement of the outcome** | **Bias due to selective reporting of the results** |  |
| **Vitamin D in blood** |  | | | | | | | |
| Skaaby, 2014 | Serious | Low | Moderate | No information | Moderate | Low | Low | Serious |
| Afzal S, 2013 | Moderate | Low | Low | Moderate | Low | Low | Low | Moderate |
| van der Pols, 2013 | Moderate | Moderate | Low | Moderate | Low | Low | Low | Moderate |
| Liang, 2012 | Moderate | Moderate | Low | Moderate | No information | Low | Low | Moderate |
| Major, 2012 | Moderate | Moderate | Low | Moderate | Low | Low | Low | Moderate |
| Tang, 2010 | Serious | Moderate | Low | Moderate | Serious | Serious | Low | Serious |
| Asgari, 2010 | Moderate | Moderate | Low | Moderate | Low | Low | Low | Moderate |
| Eide, 2011 | Serious | Moderate | Low | No information | Serious | Low | Low | Serious |
| Vojdeman, 2019 | Serious | Low | Low | No information | Low | Low | Low | Serious |
| Kwon, 2018 | Moderate | Moderate | Low | No information | No information | Low | Low | Moderate |
| **Vitamin D intake** |  | | | | | | | |
| Asgari, 2009 | Moderate | Low | Low | Moderate | Low | Low | Low | Moderate |
| Davies, 2002 | Serious | Moderate | Low | Moderate | Low | Low | Low | Serious |
| van Dam, 2000 | Moderate | Moderate | Low | Moderate | Low | Low | Low | Moderate |
| Hunter, 1992 | Moderate | Moderate | Low | Low | Low | Low | Low | Moderate |
| Park, 2016 | Moderate | Moderate | Low | Low | Low | Low | Low | Moderate |
| Kwon, 2018 | Moderate | Moderate | Moderate | No information | No information | Low | Low | Moderate |

**Supplementary Figure 1**. Flow chart

**27 546** potentially relevant publications identified

**27 262** publications excluded based on title and abstract

**85** publications included from search risk

**284** full-text articles retrieved and assessed for inclusion

**199** excluded based on the following criteria:

18 reviews or no original data

20 meta-analyses

24 case-control studies

2 other study designs

1 pooled analysis of case control studies

8 letter/editorail/commentary/magazine article

7 no exposure other than dietray factors

35 no outcome of interest

1 no measure of association

83 with at least one reason listed below

**68** publications from 2005 SLR and **37** from others searches*

**190** publications included:

157 with cohort, case-cohort or nested case-control design

28 from randomized controlled trials

5 pooled studies

**177** publications excluded : exposure others than vitamin D

**13** cohort studies included:

10 studies on circulating 25(OH)D level and skin cancer

4 studies on vitamin D intake and skin cancer

**Supplementary Figure 2.** Highest versus lowest analysis of circulating 25-hydroxyvitamin D and skin cancer. Summary estimates were calculated by using a random-effects model. A, melanoma; B, Keratinocyte cancer and C, Basal-cell carcinoma and Squamous cell carcinoma;

model.

**A**


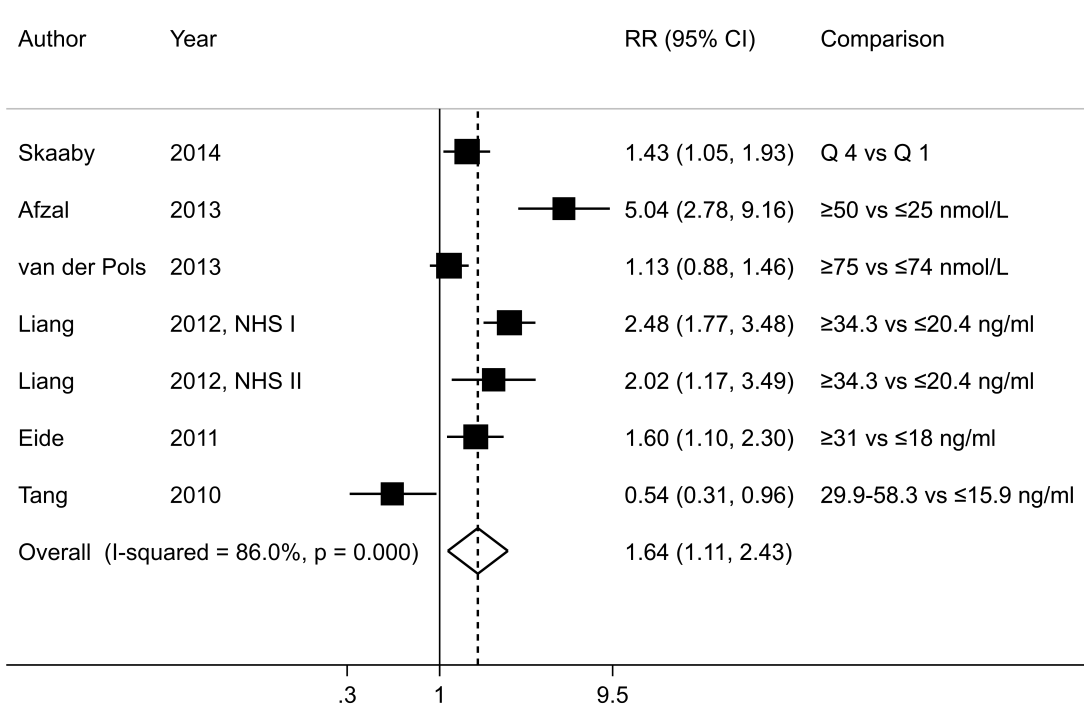

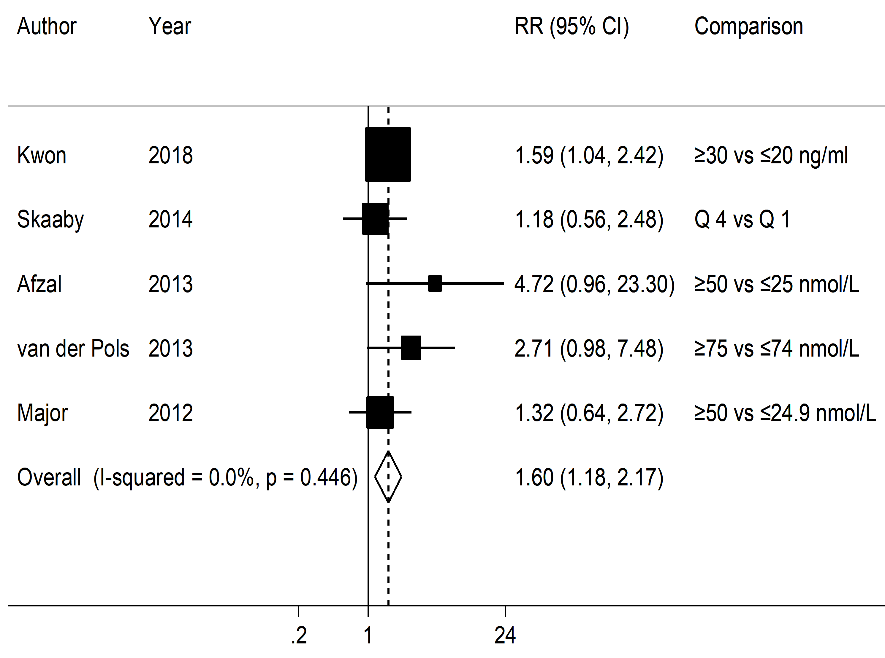


**B**

**C**


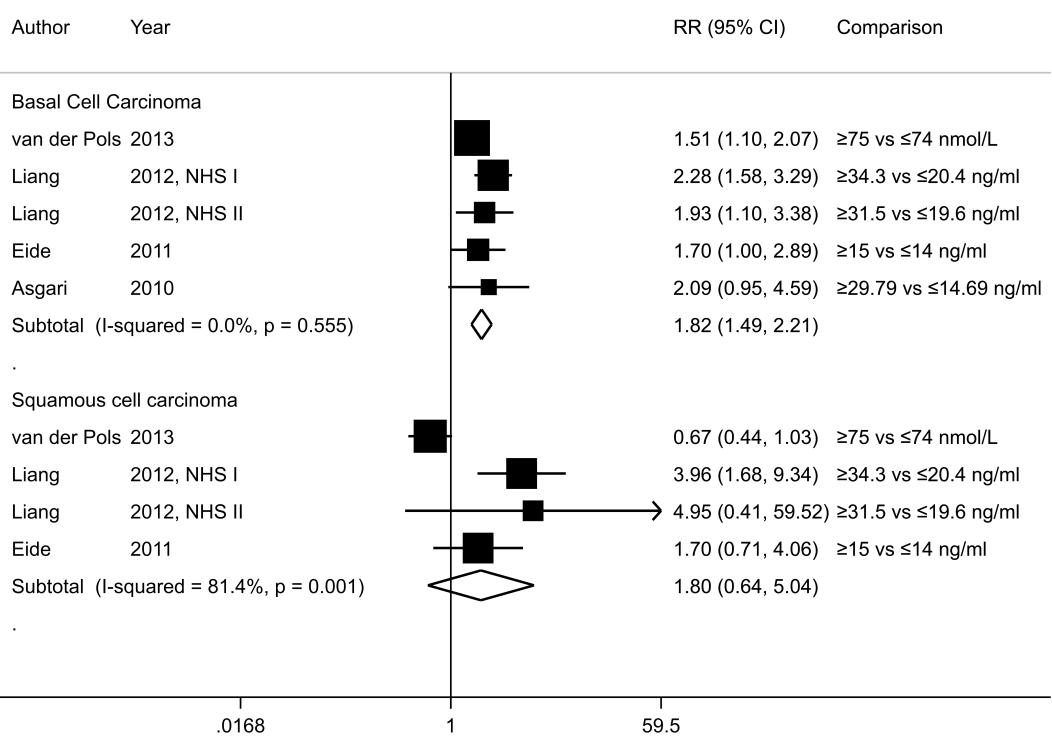


**Supplementary Figure 3**. Influence analysis of circulating 25-hydroxyvitamin D and skin cancer (for an increase of 30 nmol/L). A, melanoma and B, keratinocyte cancer.

**B**

**A**
